# Describing an old trick in posterior thoracal and lumbar ımplant removal surgery: follow-up results for at least 10 years

**DOI:** 10.1186/s40001-024-01977-3

**Published:** 2024-07-25

**Authors:** Murat Yilmaz, Ersin Ikizoglu, Onder Ertem, Mert Arslan, Serhat Resat Erbayraktar, Kemal Yucesoy

**Affiliations:** 1 Department of Neurosurgery, Faculty of Medicine, School of Medicine, Dokuz Eylul University, İnciraltı, Mithatpaşa Cd. No: 56, 35330 Balçova, Izmir Turkey; 2 Department of Neurosurgery, Faculty of Medicine, Bahcesehir University, Izmir, Turkey

**Keywords:** Thoracolumbar, Implant, Failure, Removal, Posterior

## Abstract

**Study design:**

Retrospective case series.

**Objectives:**

We aimed to describe with a novel surgical approach for the removal of posterior thoracolumbar implant in patients with symptomatic failure of the implant and present our preliminary results with this method.

**Methods:**

This retrospective, single-center study was performed in the neurosurgery department of a university hospital. Data were gathered from the medical files of 314 patients (243 women, 77.39%; 71 men, 22.61%) with symptomatic thoracolumbar implant failure that underwent implant removal operation using our novel technique between 2010 and 2020. Symptoms, radiological findings, intraoperative findings as well as clinical outcomes were evaluated.

**Results:**

In our series, the average age was 46.5 years (range: 21–84) with a mean follow-up duration of 7 years (range: 3 months to 10 years). Preoperatively, the most common symptoms were leg pain and numbness of the lower extremity. Postoperatively, no major complications were noted. Clinical progression of symptoms was avoided by surgery in all patients, while we came across removal difficulties due to screw–screwdriver mismatch in 15 of 314 surgeries (4.78%). Our novel approach allowed successful screw removal including these challenging cases.

**Conclusions:**

We suggest that our novel approach is a practical and effective for the removal of posterior thoracolumbar implant in cases with symptomatic failure attributed to screw–screwdriver mismatch. Further trials are warranted to assess the efficacy of this technique to overcome surgical problems associated with screw removal.

## Introduction

Many recent advancements in spine surgery have been fuelled by a flood of novel implant models. For many years, pedicle screw instrumentation for internal spinal column stabilization has been employed all over the World [1]. Pedicle screws may need to be removed for a variety of reasons, including screw or rod loosening/breakage with loss of fixation, adjacent segment disease, screw pull out, screw malpositioning, pseudarthrosis, surgical site infection, and adjacent segment pathology [2].

Pedicle screws are becoming more advanced as time passes and there is no universal standard size for pedicle screws among manufacturers. To put it another way, each company’s screws and other stabilizing materials, particularly key lock screwdrivers and other applicator tools, have distinct sizes and forms. Because it has been so long since modern spinal instrumentation was invented and used, procedures requiring implant removals/revisions are becoming more common. Preoperative radiographs must be used to identify implants and makers, as well as to prepare proper instruments for removal [2].

When a patient's pedicle screw-rod system needs to be altered, it is best to employ a surgical set from the same company that made the material that was used in the first surgery before the operation. However, obtaining the surgical set used in the initial surgery may not always be feasible. In this instance, significant obstacles during surgery may be encountered due to incompatibility of parts such as pedicular screws and screwdrivers, and pedicle screws may not be removed in some cases. Understanding the essential characteristics of modern spinal instruments and their radiographic appearances will aid surgeons in recognizing implants and manufacturers before surgery and will make revision procedures easier.

In the current study, we aimed to evaluate the clinical outcomes of the patients that underwent posterior thoracolumbar implant removal surgery using a novel surgical procedure and to share our experience with this method with a brief review of current literature.

## Materials and methods

### Study design

This retrospective study has been performed per the principles of the Helsinki Declaration after the approval of the local institutional review board (2020/21-06). Between 2010 and 2020, 314 patients underwent surgery for the removal of thoracic or lumbar posterior implants due to various etiologies, such as loosening of screws, adjacent segment disease, screw breakage, rod breakage, and pull-out of screws, surgery site infection, and allergic reaction.

The follow-up data were obtained from the patient charts in the hospital database or by telephone contact. All patients underwent complete preoperative diagnostic work-up, including X-ray, computed tomography (CT), and magnetic resonance imaging (MRI). The indication for surgery was implant failure attributed to various etiologies.

### Surgical technique

All patients underwent thoracolumbar posterior implant removal surgery under general anesthesia. After the skin incisions, the posterior implant system was exposed after subcutaneous tissue, fascia, and paravertebral muscle planes. If there were no screw–screwdriver mismatch, the implant removal was performed using conventional methods. Implant removal procedure was performed using our novel method in cases with the screw and screwdriver mismatch.

**Fig. 1:**
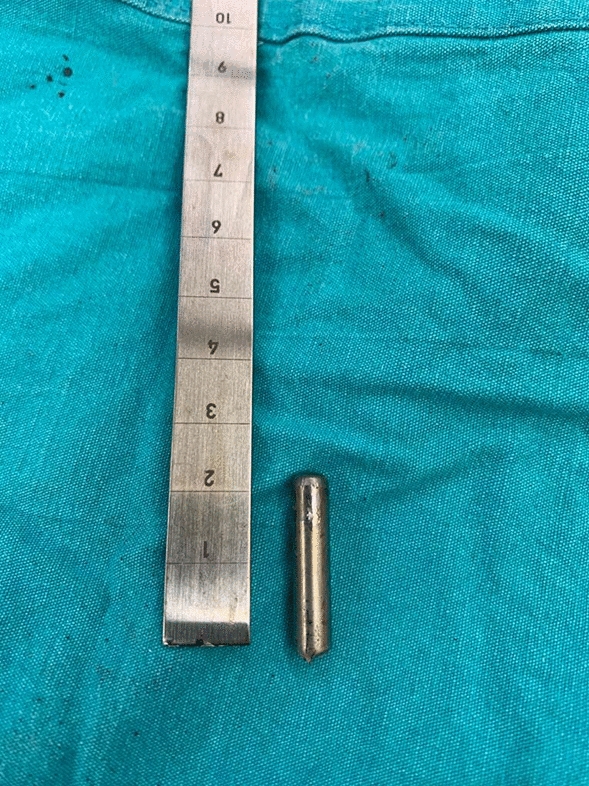
A 2 cm piece is cut from the removed rod

**Fig. 2:**
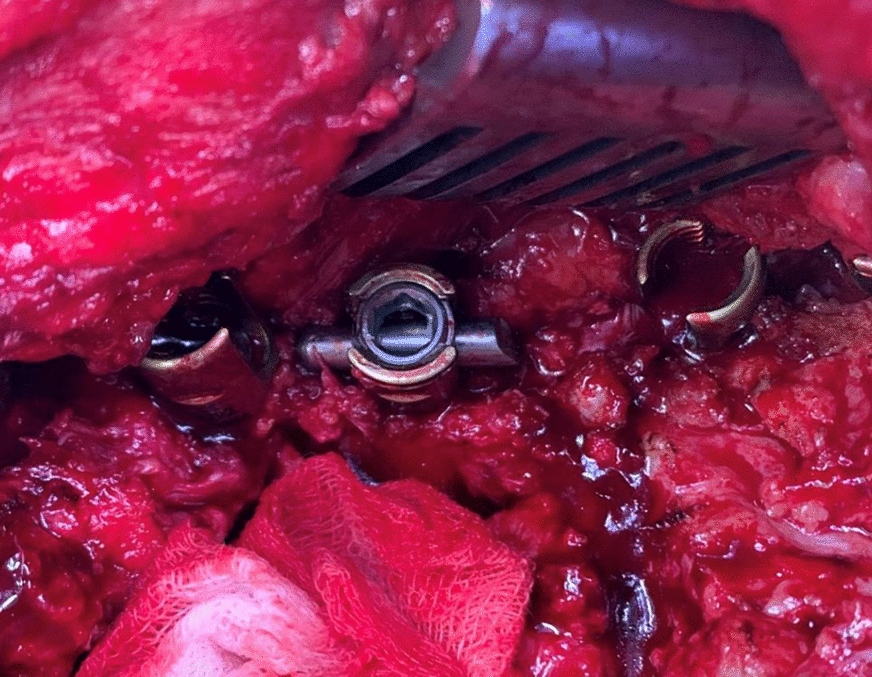
Cut piece of rod is mounted on the screw head

**Fig. 3:**
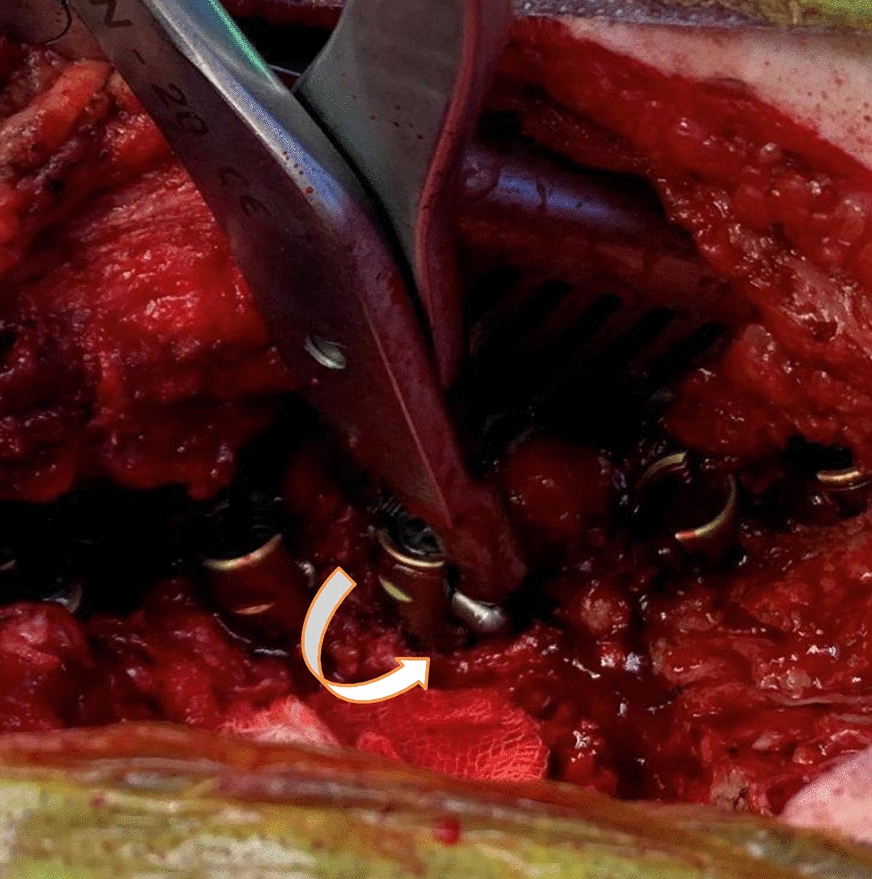
Screw, which is made into a monoblock, is removed by turning in the appropriate direction (white arrow)

**Fig. 4:**
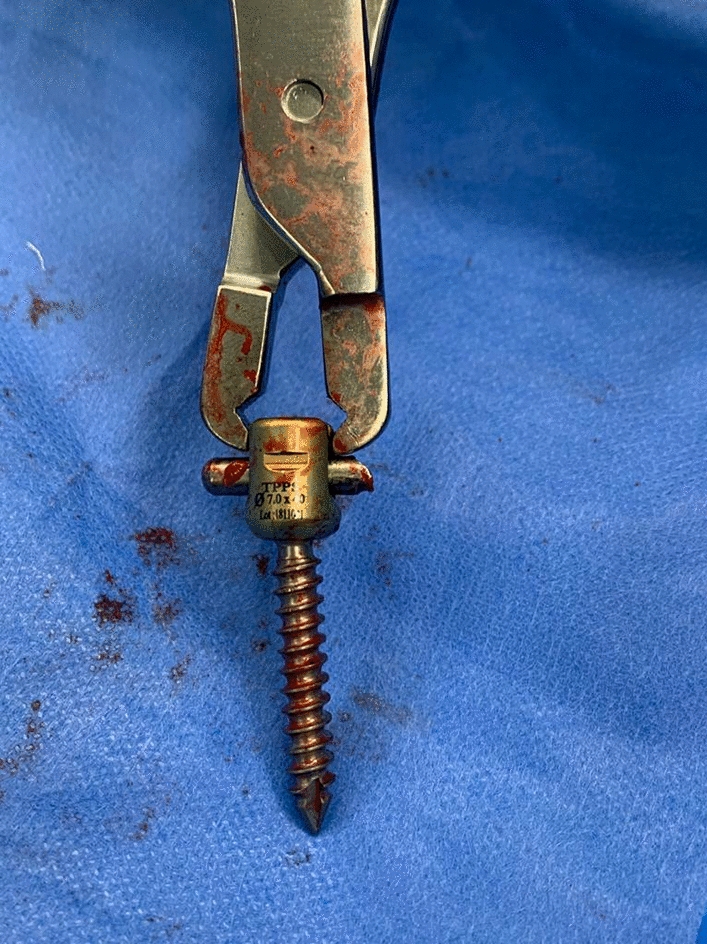
Screw is removed with tight rod holder

The screws used in posterior spinal instrumentation (PSI) surgeries are generally polyaxial and have a mobile head. It is generally not a problem to remove the nut part in the screw head, but it may sometimes be impossible to find a compatible screwdriver with a proper application channel in the screw body. In such a circumstance, a piece of approximately 2 cm is cut from the removed rod (Fig. 1) and firmly fixed on the rod to be removed using a nut (Fig. 2). Thus, the polyaxial screw is made monoaxial and the movable screw head is fixed. The fixed screw head is grasped with a strong rod holder tool and rotated in the direction of screw removal (Fig. 3). With such an application, the entire screw system can be easily removed (Fig. 4).

### Outcome parameters

Routine lumbosacral spinal MRI and CT were performed in all cases preoperatively. CT is an essential radiological examination to demonstrate the failure of the implant. The patients underwent MRI in the postoperative period, as well as at 6th and 12th months after surgery during follow-up. Visual analog scale (VAS) was used as the main outcome measure at admission and follow-up (Table 1).

**Table 1:** Results of visual analog scale at admission and follow-up

	*n*	VAS at admission	VAS at follow-up
Screw loosening	208	70	38
Adjacent segmental problem	38	80	40
Screw malposition	20	90	50
Screw pull-out	17	70	35
Screw head dislodgement	12	65	33
Screw breakage	10	64	45
Rod breakage	5	65	25

### Statistical analysis

Our data were analyzed using Statistical Package for Social Sciences program version 21.0 (*SPSS Inc., Chicago, IL; USA*). The values were expressed as mean ± standard deviation or median, minimum, and maximum.

## Results

The baseline descriptives and clinical data in our series are displayed in Table 2. Our population consisted of 71 men and 243 women with a mean age of 46.5 years (range 21–84). The mean follow-up was 7 (range, 3 months to 10 years) years. 208 patients had screw loosening, 38 had adjacent segment problem, 20 had screw malposition, 17 had screw pull out, 12 had screw head stripping, 10 had screw breakage, 5 had rod breakage, 3 had surgical site infection and one patient had titanium allergy.

**Table 2:** Baseline descriptives and clinical findings in our series

Variable		
Sex		
Female	243 (77.39%)	
Male	71 (22.61%)	
Age (mean, range)	46.5 years	21–84 years
Duration of follow-up (mean, range)	7 years	3 months–10 years
Indication for implant removal		
Screw loosening	208 (66.24%)	
Adjacent segmental problem	38 (12.10%)	
Screw malposition	20 (6.37%)	
Screw pull-out	17 (5.41%)	
Screw head dislodgement	12 (3.82%)	
Screw breakage	10 (3.18%)	
Rod breakage	5 (1.59%)	

**Fig. 5:**
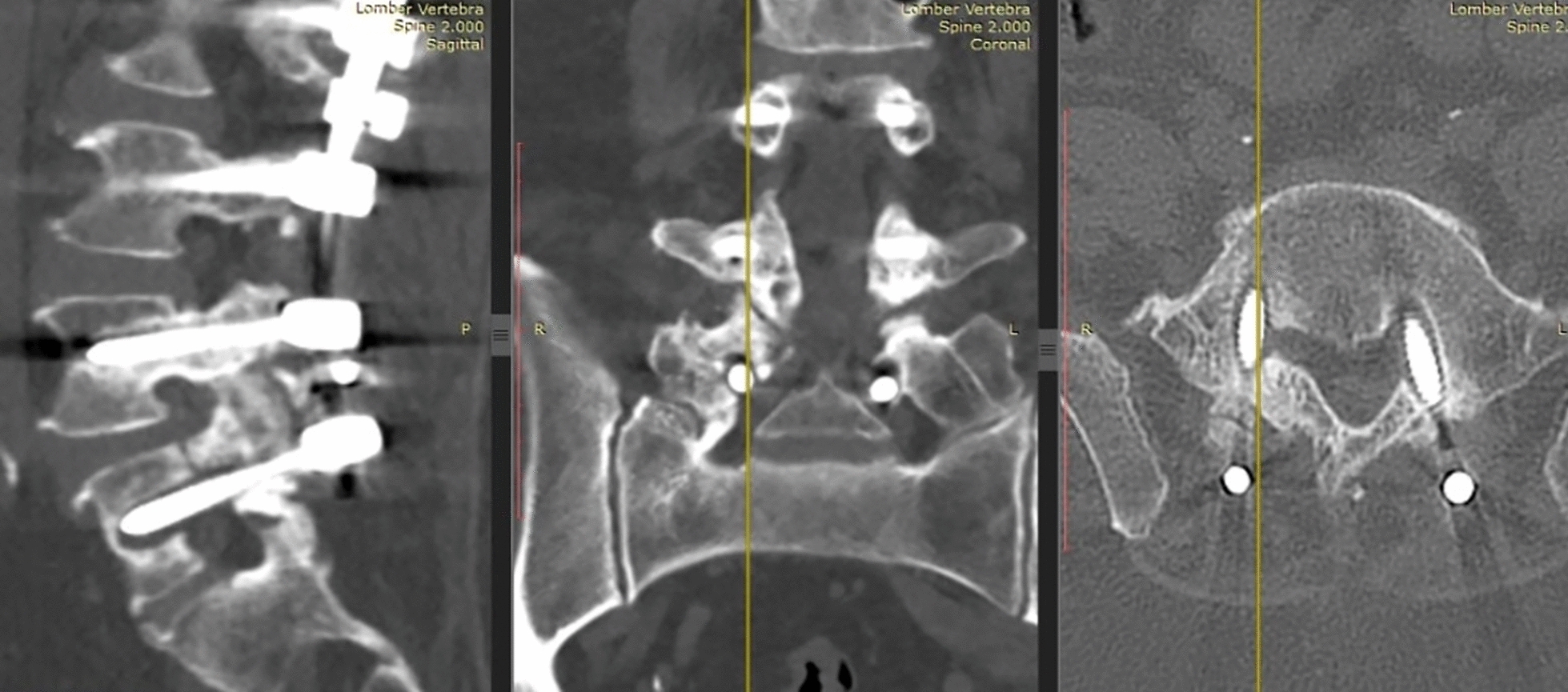
Axial, coronal and sagittal views demonstrate screw loosening on CT scans

**Fig. 6:**
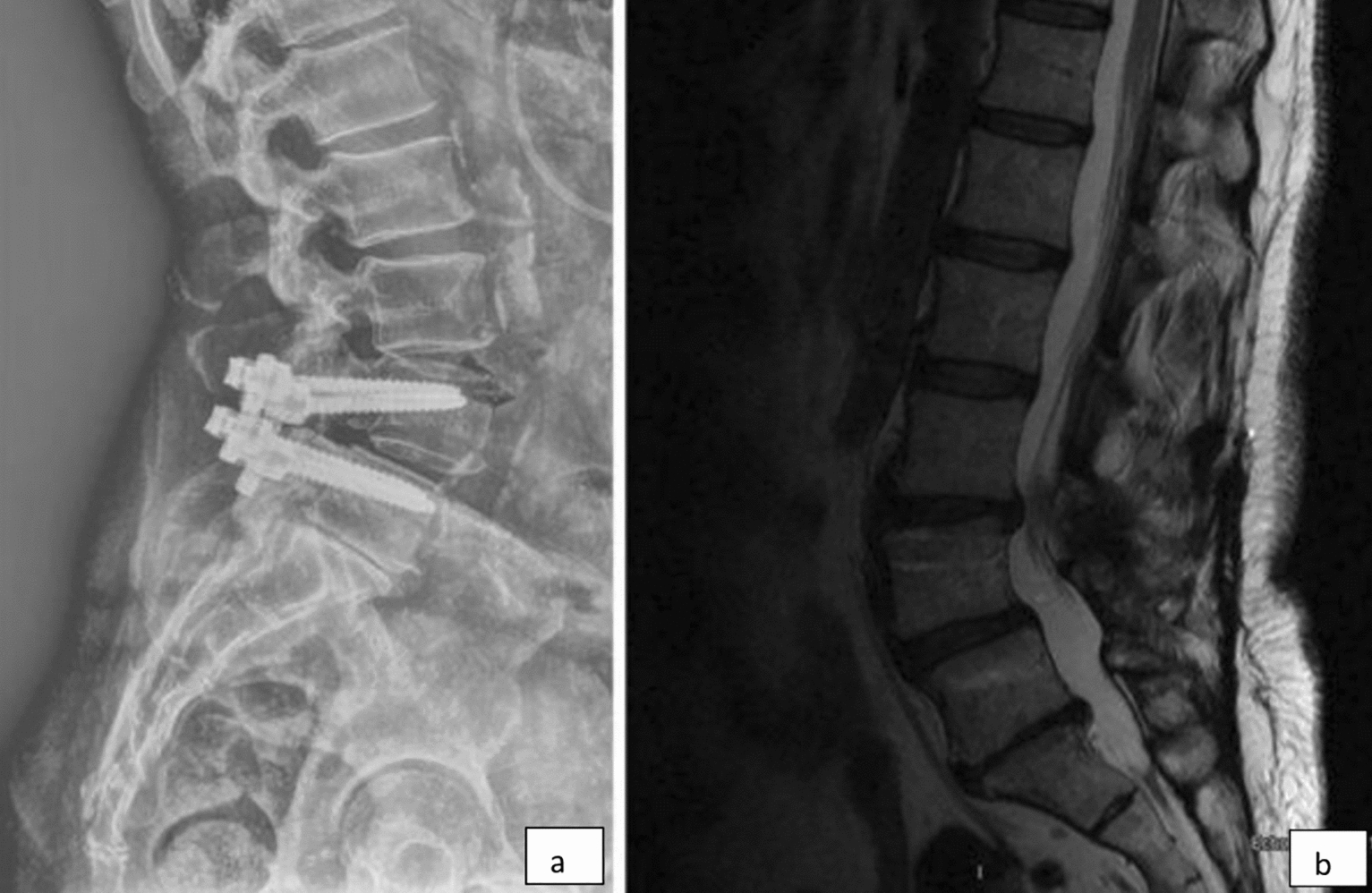
**a**, **b** Lateral X-ray and sagittal views of MRI demonstrating adjacent segment disease

**Fig. 7:**
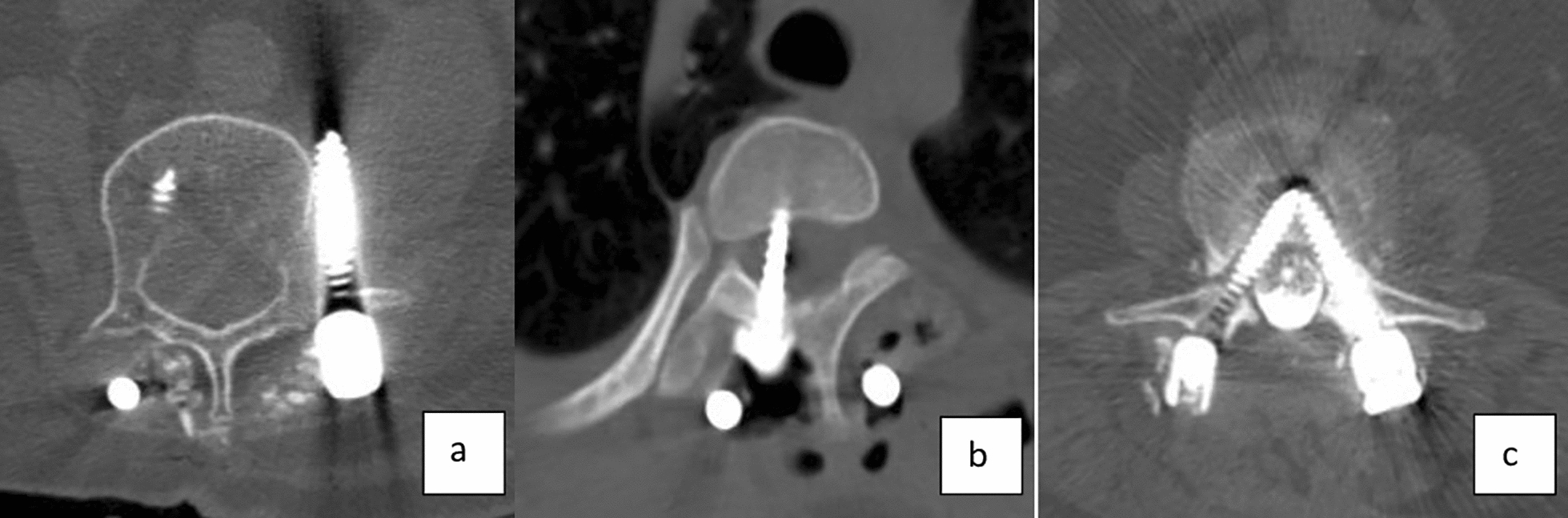
**a**–**c** Axial CT scans demonstrating screw malposition

**Fig. 8:**
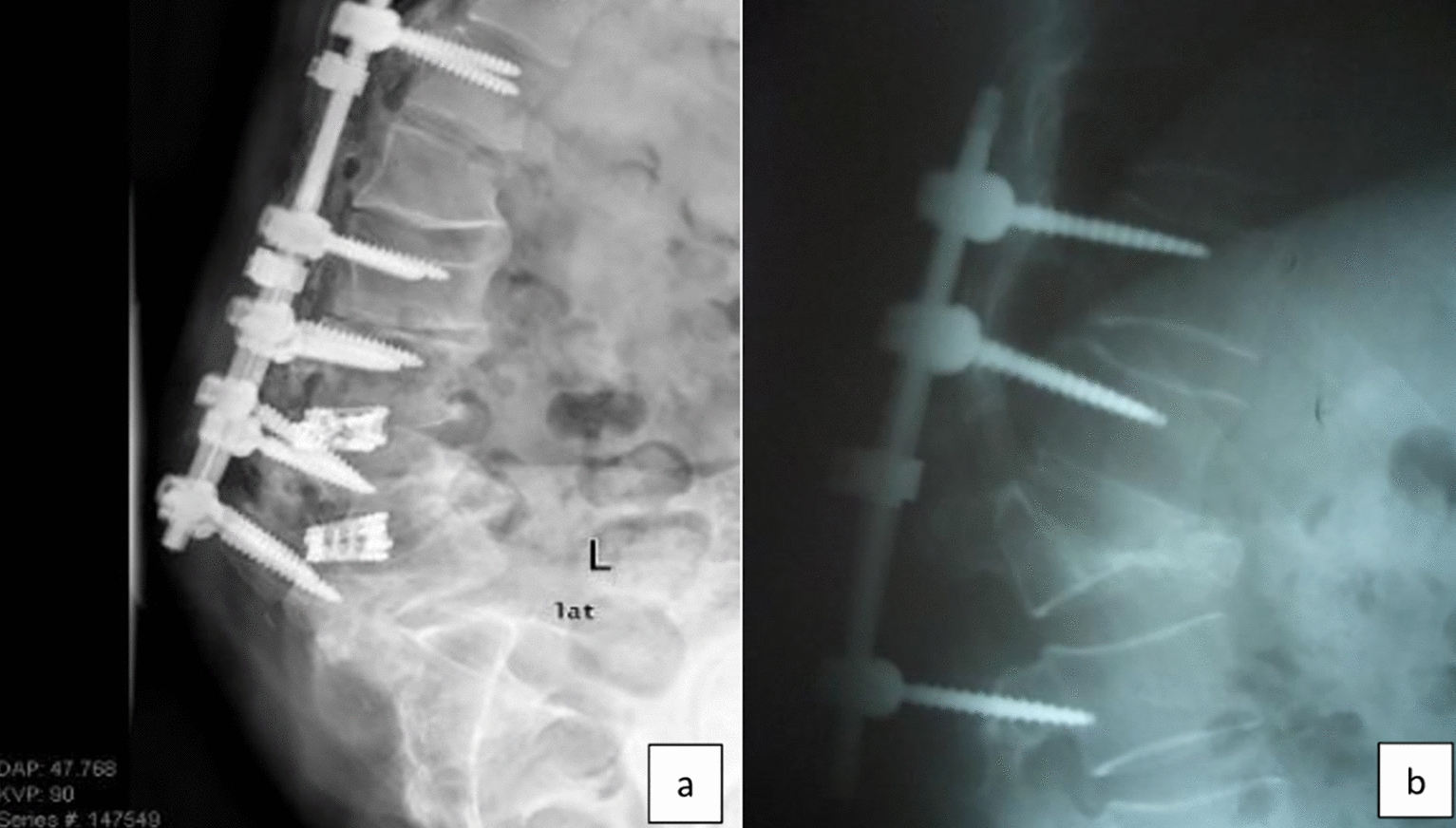
**a**, **b** Lateral X-ray shows screw pull out

**Fig. 9:**
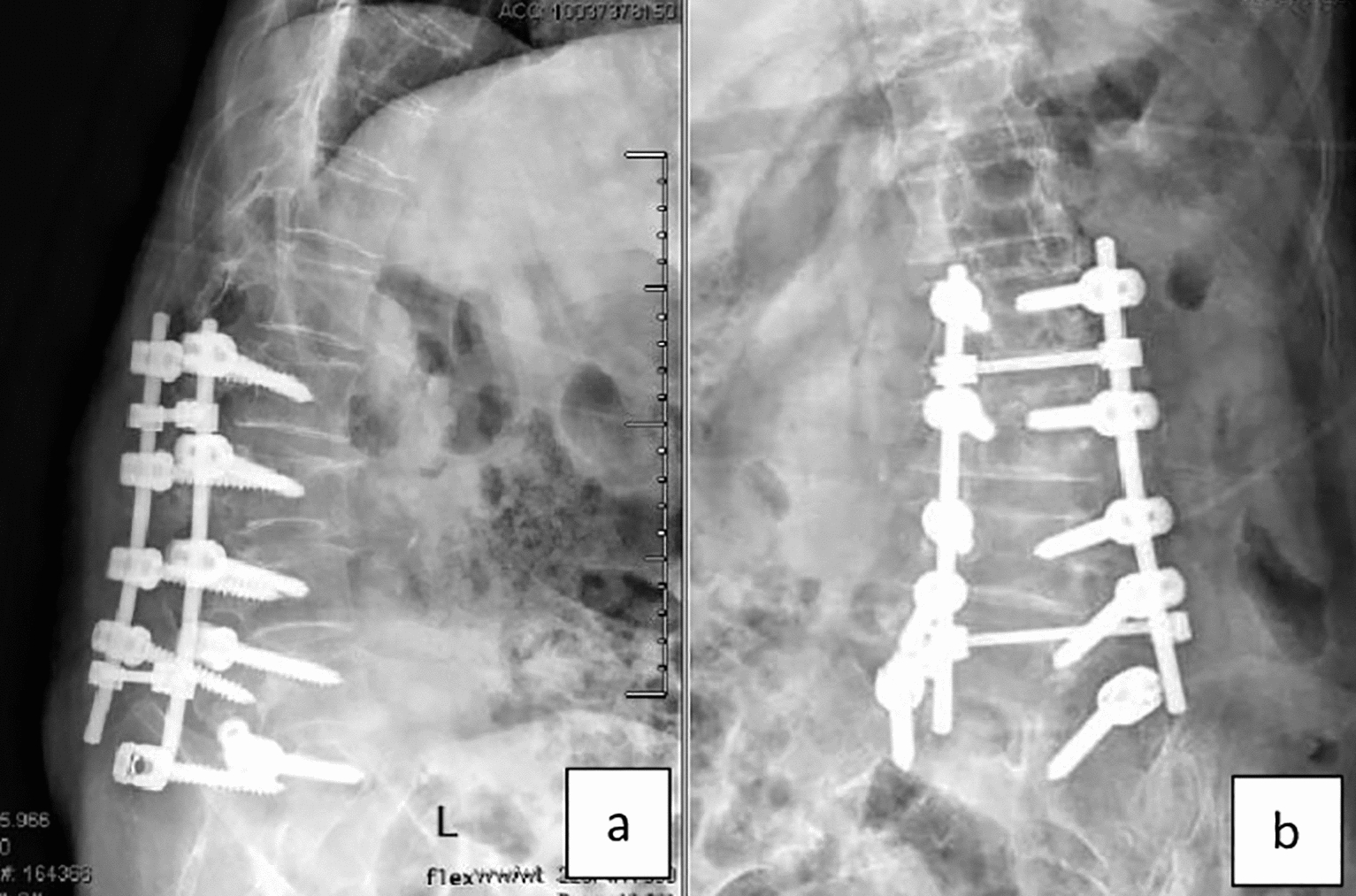
**a**, **b** Lateral X-ray shows screw head opening

**Fig. 10:**
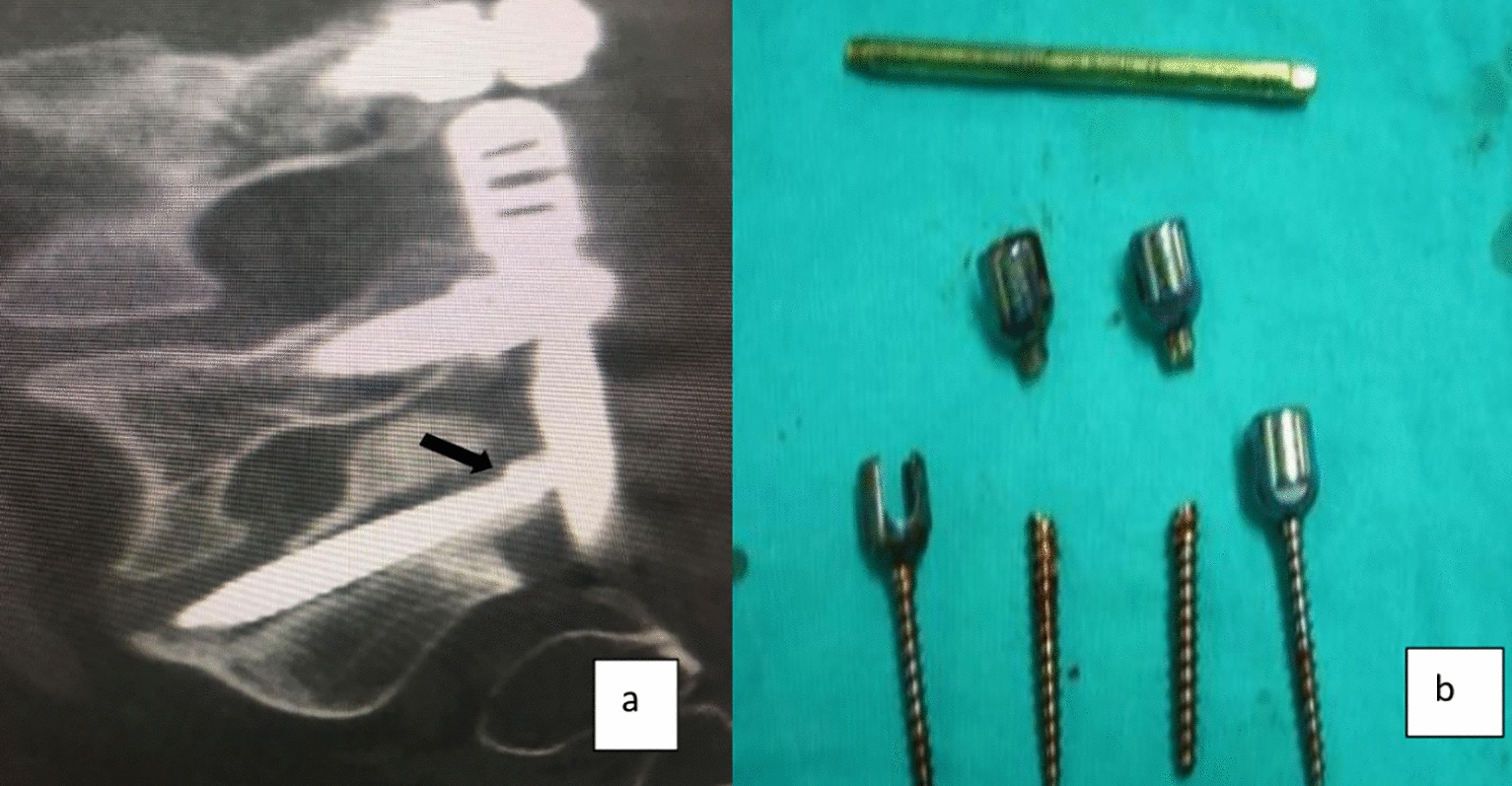
**a**, **b** Sagittal CT scans (black arrow) and image demonstrating screw breakage

**Fig. 11:**
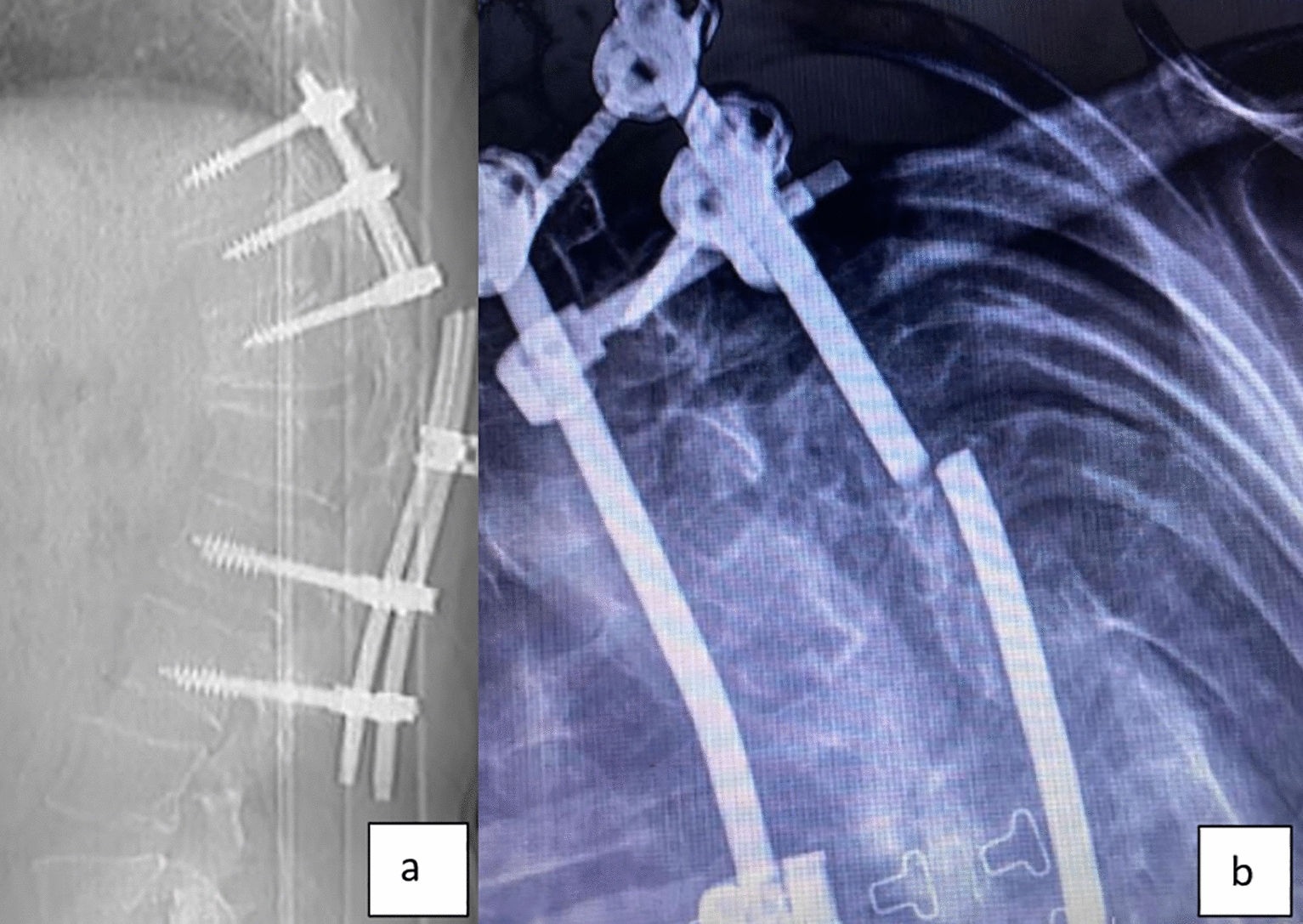
**a**, **b** Lateral and anteroposterior X-ray images demonstrating rod breakage

Main problems encountered in our population were loosening of the screw (*n* = 208) (Fig. 5), repositioning the system due to adjacent segmental problem (*n* = 38) (Fig. 6), screw malposition (*n* = 20) (Fig. 7), screw pull-out (*n* = 17) (Fig. 8), screw head dislodgement (*n* = 12) (Fig. 9), screw breakage (*n* = 10) (Fig. 10), rod breakage (*n* = 5) (Fig. 11), infection at the surgical site (*n* = 3), and allergic reaction against titanium (*n* = 1).

Patients with infection (*n* = 3) and allergy (n = 1) were excluded from the study. Therefore, data gathered from a total of 314 patients were analyzed.

The removal of the PSI in thoracic, thoracolumbar, and lumbosacral regions was performed in 5, 27, and 282 patients, respectively. No patients underwent removal of the posterior cervical system. The most common cause of system disassembly was screw loosening. The average decrease in VAS for pain after implant removal was statistically significant (from 72 to 38, *p* < 0.001).

In 15 cases (4.78%), removal of the implant was challenging attributed to screw–screwdriver mismatch. In these patients, our novel method facilitated the removal of the implant. Postoperatively, no major complications were detected. Clinical progression of the symptoms was successfully halted by the removal of the implant in all patients. None of the systems removed were dynamic. Therefore, the fusion rates are supposed to be satisfactory. Fusion rates were examined by CT scans. The implant removal was not performed 3 months after the initial surgery, except for the 3 patients with infection. A period of 3–4 months was sufficient for the achievement of bone fusion.

For 71 patients who had their initial surgeries in other medical centers, the performance of bone fusion could not be confirmed. For procedures performed in our center, bone fusion (autograft, allograft, or combined) was aimed during the first surgery. In addition to posterior stabilization, the posterior lomber interbody fusion (PLIF) procedure was employed in 15 patients. Intraoperatively, 15 patients did not display solid fusions. In ten of these patients, the system was renewed with a cannulated screw system reinforced with methylmethacrylate, and the fusion was strengthened in 5 of them with only 20 g of allograft bone fusion material. Grade 2 spondylolisthesis was observed postoperatively in one of the patients whose system was completely removed, and fractures in the adjacent vertebrae were observed in three cases. These four patients were taken to surgery again and PSI was performed.

All the patients were mobilized using thoracolumbosacral orthosis on the next day after surgery. Plain radiographs and CT scans were obtained routinely after surgery.

No additional neurological deficits developed in any of the patients in the postoperative period. Postoperative infection that occurred in two patients resolved completely in response to appropriate antibiotic therapy. The indication for implant removal was “implant-associated pain” in all cases. The mean preoperative VAS for pain was 7, 2, ranging from 1 to 10.

## Discussion

The removal of the pedicle screw tool is usually considered a simple and benign process in spinal surgical procedures. Implant removal may alleviate worries about the dangers of metal indwelling, such as micromotion, metal fretting, allergic reaction, infection, or stress-induced osteopenia [3, 4]. Furthermore, removing the pedicle screw reduces the rigidity of the fused segment, which may reduce stress concentration at surrounding segments [5, 6]. Pedicle screw removal, on the other hand, is a second surgical procedure conducted under general anesthesia, with associated morbidity concerns such as operative site infection, neurovascular damage, and refracture.

Problems associated to the construct used in spinal deformity surgery are common. Loosening/breakage of the implant with loss of fixation, pseudarthrosis, neighboring segment disease, and surgical site infection are all common clinical situations. Revision procedures are frequently required as a result of these problems. Revision operations are becoming more common as time has passed since these systems were introduced. The removal or replacement of prior implants, with or without the addition of new implants, may be included in such revision operations. Some commonly used implants may be removed with universal screwdrivers, whereas other systems require the use of specialised equipment. It's crucial to know which implant systems were employed in earlier procedures. Despite the vast range of implant designs in use, information regarding the precise implant utilized are not always noted in operative reports, and in some cases, operative reports are not even available, such as when surgeries are performed in remote locations or revisions are required on an emergency basis. As a result, a preoperative radiographic evaluation of the prior construct is required as part of the preparation process [2].

The rates of posterior thoracolumbar implant removal are mostly unknown. It's also debatable how much assistance patients receive. Only 12% of individuals who have had their implants removed will experience complete symptom remission. Patients who will have implants removed should be reminded not to have unrealistic expectations, according to the same article. In many circumstances, however, the removal of spinal implants is unavoidable [1]. In contrary to literature data, pain alleviation was observed in 290 of 314 patients (92.35%) in the early postoperative period of 2 weeks [7]. However, during the postoperative 3rd month follow-up, 45 patients reported partial recurrence of pain. All patients were advised to wear a steel underwire corset in the first 2 months after PSI removal.

In case of need for iimplant removal, challenges may arise due to tool incompatibility during the procedure. The removal procedure we have described seems to be practical and safe yielding satisfactory outcomes. Before performing this novel procedure, we used to end the intervention without removal of the implants. We suggest that this method can be simply and successfully employed to any posterior screw-rod systems.

“Orthopedically damaged screw removal sets” are offered according to recent marketing brochures distributed by a spinal instrument manufacturer. Only a 2 cm section of the posterior spinal rod that we cut during surgery plays a critical role in the effectiveness of the surgery. Thus, there is no need to employ sets including a huge number of newly released instruments.

Implant removal after successful fusion was advantageous in our present study with thoracolumbar burst fractures because it greatly reduced pain and impairment. The exact process by which clinical improvement occurs following implant removal is unknown. Implant-related pain has been linked to micromotion, metal fretting, allergic reaction, low-grade infection, and/or stress concentration in the neighboring segment [5, 6, 8]. Implant removal and fusion exploration may be an acceptable therapy to try to reduce pain in patients who report with recurrent persistent back pain and no other identifiable causes shown to be pain generators. However, there is scarce data on the implications of removal of hardware following instrumented spinal fusions.

The removal of spine implants for pain management in solid fusion patients has been a point of contention. The relevance of spinal implant removal in the therapy of prolonged pain and symptoms following spinal fusion remains unclear. The exact method by which the implant causes discomfort is still unknown. The rationale and outcomes of removing a spinal implant in terms of pain management are unknown. The removal of equipment is typically seen to be a reasonably benign process, despite the fact that the outcome appears to be uncertain. As a result, neurosurgeons will consider removing equipment to help patients with their problems [9]. The morbidity associated with undergoing an additional surgery is a final factor when evaluating the value of implant removal. Perioperative risk varies from patient to patient, but this adds to the complexity of the surgeon–patient clinical decision [7].

Aono et al. [10] claim that using titanium as a screw material reduces the chance of early instrument fixation failure because the material used can affect the risk of failure. Titanium has two times the strength and flexibility of typical stainless steel [11]. According to another study, utilizing pedicle screws with the largest diameter possible can also help prevent failure [12].

Implant removal is supposed to be a safe procedure with satisfactory outcomes with improvement of quality of life [13]. Majority of patients report benefit from removal of the implant [13]. We hope our novel technique can be a safe, cost-effective, and practical method in selected patients scheduled for removal.

The main restrictions of the present paper include retrospective and single-center design, variability of outcomes with different implant brands. Moreover, the implant removal was not performed in a completely random manner and inclusion of study subjects may result in selection bias. The cost-effectiveness of implant removal procedure was not assessed and the decision to remove pedicle screws has significant financial ramifications, given the costs of the treatment under general anesthesia as well as time off for postoperative rehabilitation. Other social, economic, and psychosocial elements that could have influenced clinical results must be remembered during extrapolation of our data to larger populations. More research is needed to understand if any of these characteristics influence the clinical outcome in patients with healed thoracolumbar burst fractures after pedicle screw removal.

## Conclusion

Although the benefits and grounds for implant removal are debatable, the need for this intervention is not rare. In individuals with back discomfort after spine surgery, a retained implant could be one of the pain generators. For properly selected patients with implant-related discomfort, implant removal may give significant pain relief and be a safe treatment. The results of the present study demonstrated that understanding the radiographic characteristics of earlier spinal implants would aid surgeons in preparing for the rising number of revision surgery. This screw removal technique we have developed is effective and reasonable to use in cases with screw–screwdriver mismatch in posterior thoracolumbar implant removal surgery. Even though we mainly focused on thoracolumbar spine implant removal surgery, the same concept can be employed to cervical and sacral spine implant removal surgeries. Validation of our results necessitate implementation of further randomized, controlled trials on larger series.
